# [Retracted] MicroRNA‑106a‑5p promotes the proliferation, autophagy and migration of lung adenocarcinoma cells by targeting LKB1/AMPK

**DOI:** 10.3892/etm.2024.12603

**Published:** 2024-06-11

**Authors:** Yushan Zhou, Yuxuan Zhang, Yanli Li, Liqiong Liu, Zhidong Li, Yanhong Liu, Yi Xiao

Exp Ther Med 22:1422, 2021; DOI: 10.3892/etm.2021.10857

Following the publication of this paper, it was drawn to the Editors’ attention by a concerned reader that certain of the Transwell migration assay data shown in Fig. 2D and 4F, and the cell proliferation assay data shown in Fig. 2C and 4E, on p. 4 and 6 were strikingly similar to data appearing in different form in other articles written by different authors at different research institutes that had already been published elsewhere prior to the submission of this paper to *Experimental and Therapeutic Medicine* (some articles of which have already been retracted). In view of the fact that the abovementioned data had already apparently been published previously, the Editor of *Experimental and Therapeutic Medicine* has decided that this paper should be retracted from the Journal. The authors were asked for an explanation to account for these concerns, but the Editorial Office did not receive a reply. The Editor apologizes to the readership for any inconvenience caused.

